# Supplementary material for: Ecological plasticity governs ecosystem services in multilayer networks
Source: Commun Biol. 2021 Jan 18;4:75. doi: 10.1038/s42003-020-01547-3 (PMC7813848; doi:10.1038/s42003-020-01547-3)
Supplement: Supplementary file 2 — Supplementary Material [file 42003_2020_1547_MOESM2_ESM.pdf]

Supplementary Information

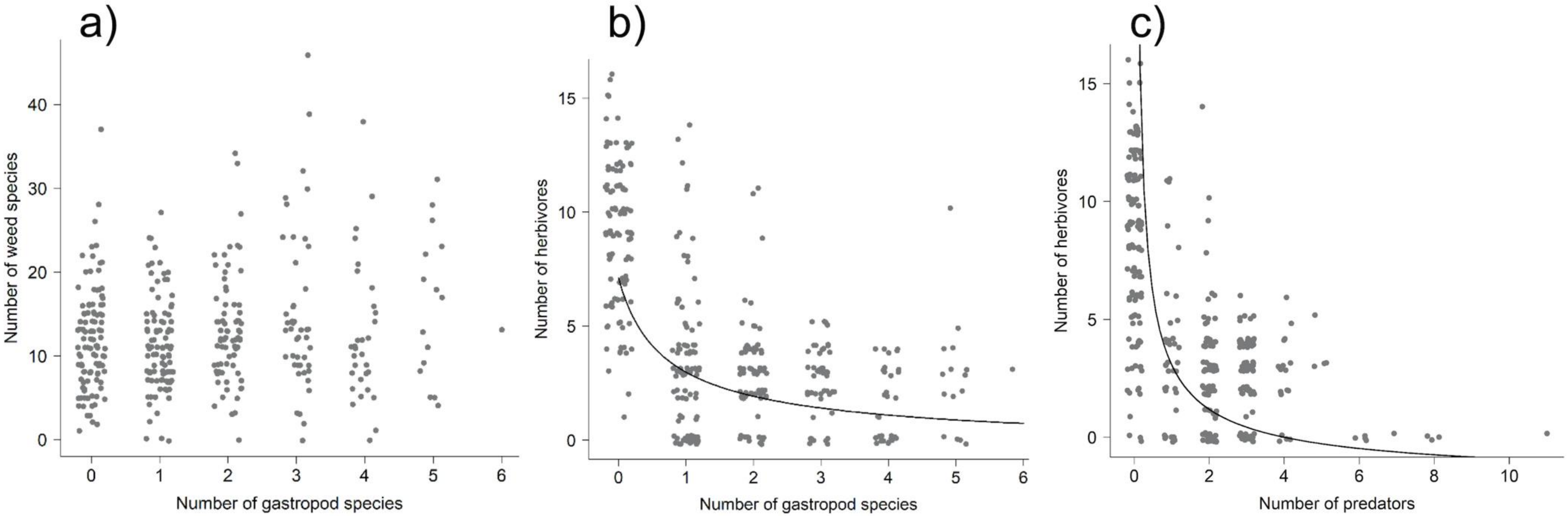

Figure S1. The relationships between carabid - weed seed and carabid – gastropod network variables. a) the number of gastropod and weed species in each network; **b)** the richness of gastropod species and the number of carabid species acting as herbivores of weed seed in each network; and, **c)** the number of carabid species acting as predators of gastropods and the number of carabid species acting as herbivores of weed seed in each network. Lines in b) and c) are regressions from GLMMs (Methods, Table S1).

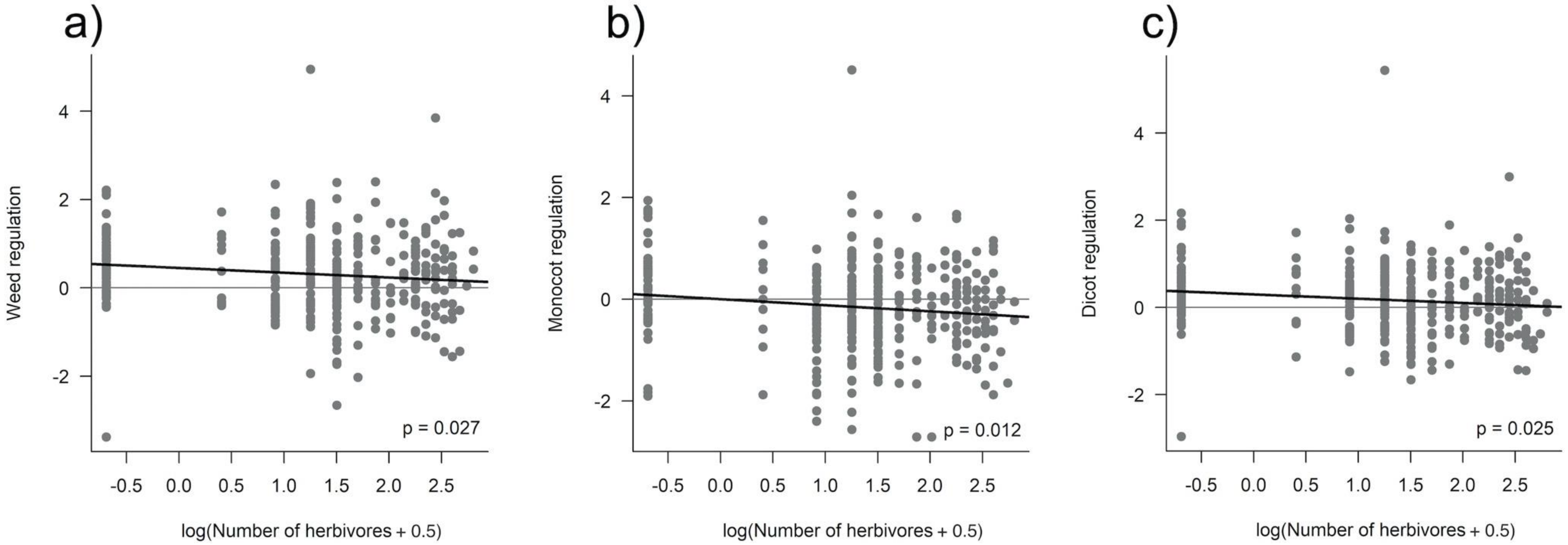

Figure S2. The level of weed seed regulation in relation to the log-transformed abundance of carabids acting as herbivores. a) the regulation of the summed total of weed seeds; **b)** monocotyledon regulation; and, **c)** dicotyledon weed seed regulation. Solid lines in a), b) and c) are regressions from GLMMs (Methods, Table S1). The line at 0 indicates the threshold where weed seedbanks decline between t_0 and t_1.

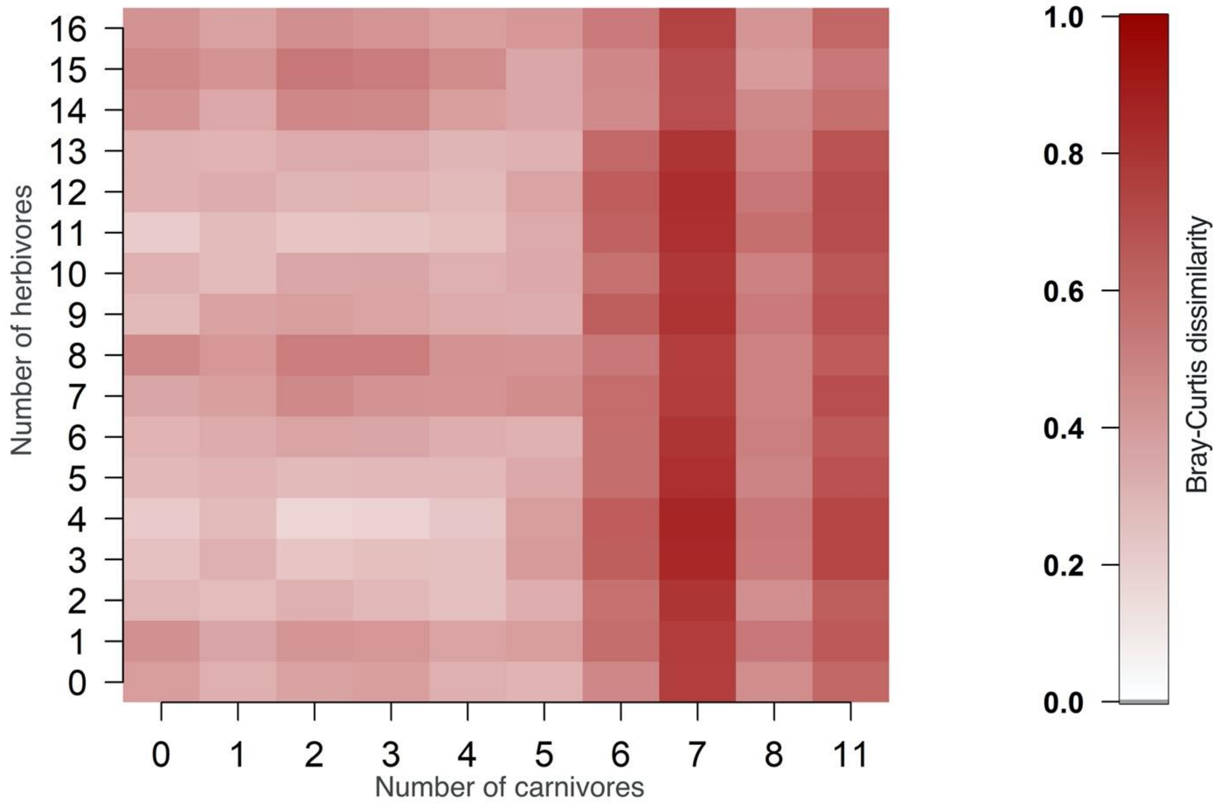

Figure S3. The estimated turnover in carabid species between networks, as Bray-Curtis dissimilarity, across the herbivore/carnivore gradient.

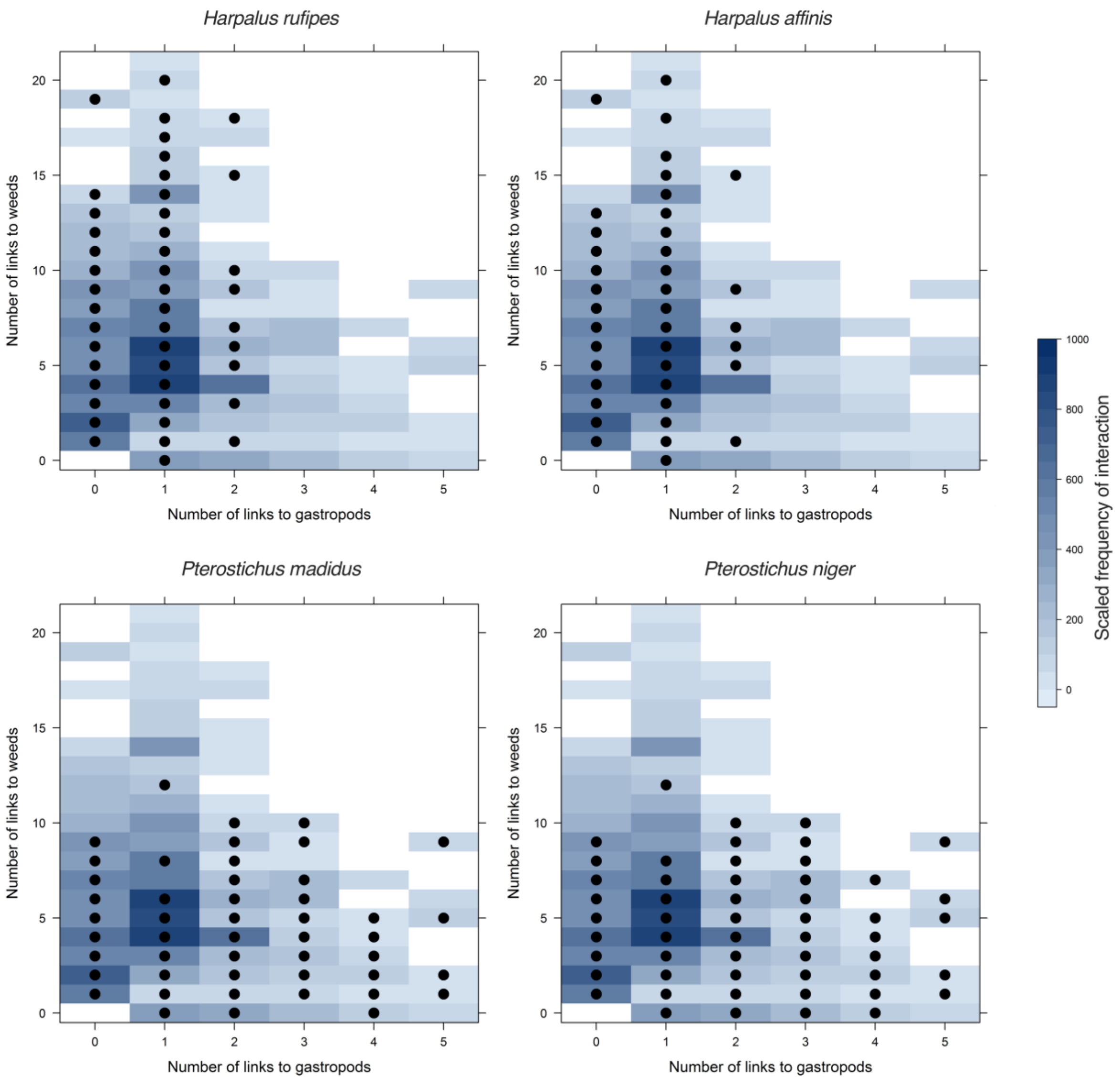

Figure S4. A density plot showing the trade-off in herbivore links and carnivore links. Within each network, for each carabid species, the number of links to weed and gastropod resources are plotted. Some carabids were pure herbivores or carnivores, but most were omnivores. Colour indicates the frequency of each particular weed-gastropod link combination, across all networks, scaled between 0 and 1000. The occupancy of this space of potential feeding interactions for the four most common carabid species after *Pterostichus melanarius*, which is shown in Fig. 4, is shown here as black circles.

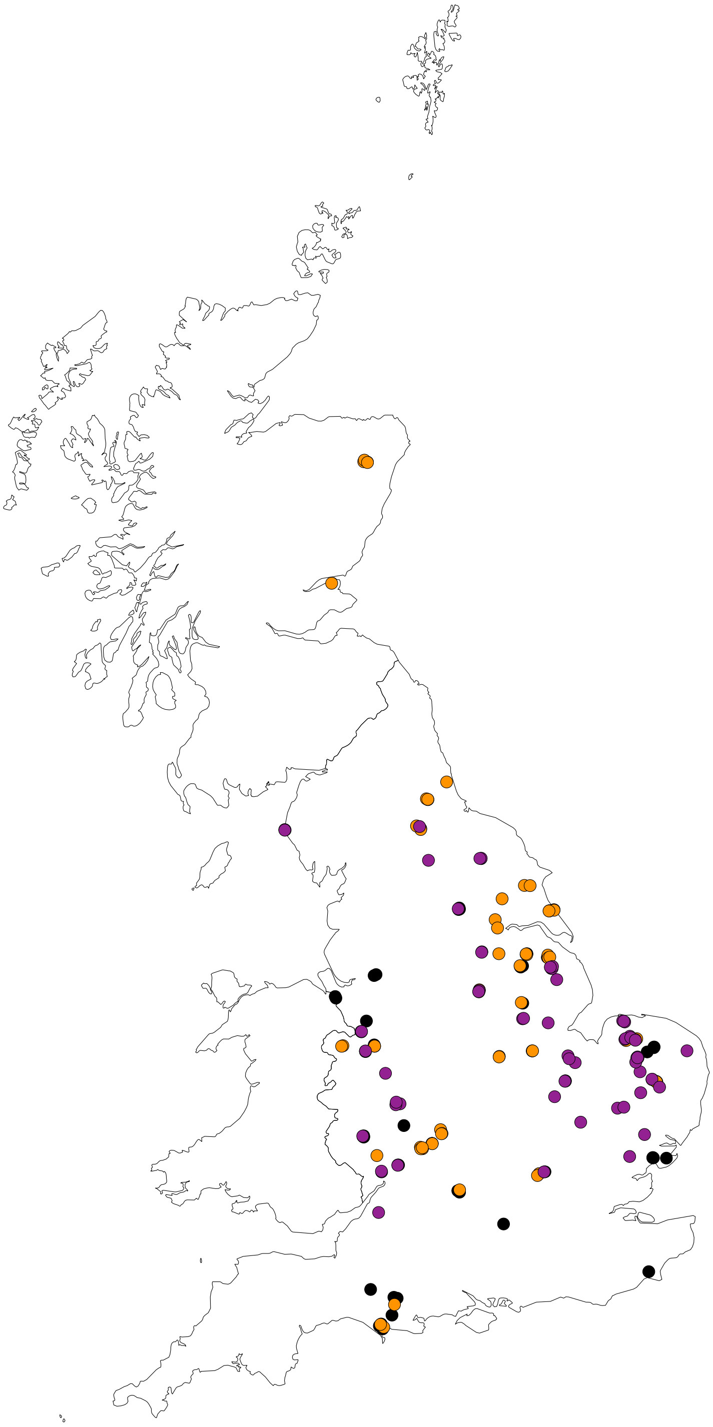

Figure S5. Map of the location of each field site sampled in the Farm Scale Evaluations (FSE) of genetically modified, herbicide-tolerant crops. The filled circles represent the three spring-sown crops trialled in the FSEs, with purple circles being the spring-sown beet sites, orange circles being those of maize and the black circle being the oilseed rape (canola) fields. The map was created using Magic Maps.

Table S1. Statistics of fit for the multiple Generalised linear mixed effects models discussed in the main text. In all models, field identity was nested within crop type (spring-sown beet, spring maize or spring oilseed rape), which was nested within management type (conventional or GMHT) and included as a random effect.

Explanatory variable	Response variable	F statistic	p-value	Figure
Species richness				
log (number of gastropod species + 0.5)	log (number of weed species + 0.5)	$F_{1,367} = 0.071$	0.7904	S1a
log (number of gastropod species + 0.5)	log (number of herbivores + 0.5)	$F_{1,367} = 182.1$	<0.0001	S1b
log (number of predators + 0.5)	log (number of herbivores + 0.5)	$F_{1,367} = 212.8$	<0.0001	S1c
Link richness				
Number of links to gastropods	Number of links to weeds	$F_{1,1334} = 356$	<0.0001	3b
Weed regulation				
log (number of herbivores + 0.5)	Total weed regulation	$F_{1,328} = 4.9$	0.0275	S2a
log (number of herbivores + 0.5)	Monocot weed regulation	$F_{1,328} = 6.4$	0.0117	S2b
log (number of herbivores + 0.5)	Dicot weed regulation	$F_{1,328} = 5.1$	0.0246	S2c
log (herbivore interaction frequency + 0.5)	Total weed regulation	$F_{1,328} = 5.2$	0.0227	2a
log (herbivore interaction frequency + 0.5)	Monocot weed regulation	$F_{1,328} = 3.9$	0.0493	2b
log (herbivore interaction frequency + 0.5)	Dicot weed regulation	$F_{1,328} = 5.72$	0.0173	2c
