# Supplementary material for: Ecological plasticity governs ecosystem services in multilayer networks
Source: Commun Biol. 2021 Jan 18;4:75. doi: 10.1038/s42003-020-01547-3 (PMC7813848; doi:10.1038/s42003-020-01547-3)
Supplement: Supplementary file 3 — Reporting Summary [file 42003_2020_1547_MOESM3_ESM.pdf]

nature research

Corresponding author(s): David A. Bohan

Last updated by author(s): Oct 21, 2020

Reporting Summary

Nature Research wishes to improve the reproducibility of the work that we publish. This form provides structure for consistency and transparency in reporting. For further information on Nature Research policies, see our Editorial Policies and the Editorial Policy Checklist.

Statistics

For all statistical analyses, confirm that the following items are present in the figure legend, table legend, main text, or Methods section.

n/a	Confirmed	
☐	☒	The exact sample size (n) for each experimental group/condition, given as a discrete number and unit of measurement
☐	☒	A statement on whether measurements were taken from distinct samples or whether the same sample was measured repeatedly
☒	☐	The statistical test(s) used AND whether they are one- or two-sided<br>*Only common tests should be described solely by name; describe more complex techniques in the Methods section.*
☐	☒	A description of all covariates tested
☐	☒	A description of any assumptions or corrections, such as tests of normality and adjustment for multiple comparisons
☐	☒	A full description of the statistical parameters including central tendency (e.g. means) or other basic estimates (e.g. regression coefficient) AND variation (e.g. standard deviation) or associated estimates of uncertainty (e.g. confidence intervals)
☐	☒	For null hypothesis testing, the test statistic (e.g. F, t, r) with confidence intervals, effect sizes, degrees of freedom and P value noted<br>*Give P values as exact values whenever suitable.*
☒	☐	For Bayesian analysis, information on the choice of priors and Markov chain Monte Carlo settings
☒	☐	For hierarchical and complex designs, identification of the appropriate level for tests and full reporting of outcomes
☒	☐	Estimates of effect sizes (e.g. Cohen's d, Pearson's r), indicating how they were calculated

Our web collection on statistics for biologists contains articles on many of the points above.

Software and code

Policy information about availability of computer code

Data collection	The data were collected as part of a large scale analysis of the effects of Genetically Modified, herbicide-tolerant (GMHT) crops in the UK. These data were published and peer reviewed as part of a number of papers in the period 2003-2006. A special issue of Philosophical Transactions of the Royal Society (Issue 358, 2003) collated much of the work and description of the data.
Data analysis	The data analysis conducted in this paper was done using open source statistical tools and published methods, as described in the text.

For manuscripts utilizing custom algorithms or software that are central to the research but not yet described in published literature, software must be made available to editors and reviewers. We strongly encourage code deposition in a community repository (e.g. GitHub). See the Nature Research guidelines for submitting code & software for further information.

Data

Policy information about availability of data

All manuscripts must include a data availability statement. This statement should provide the following information, where applicable:

- Accession codes, unique identifiers, or web links for publicly available datasets
- A list of figures that have associated raw data
- A description of any restrictions on data availability

A data and materials statement is provided in the manuscript.

Field-specific reporting

Please select the one below that is the best fit for your research. If you are not sure, read the appropriate sections before making your selection.

☐ Life sciences ☐ Behavioural & social sciences ☒ Ecological, evolutionary & environmental sciences

For a reference copy of the document with all sections, see nature.com/documents/nr-reporting-summary-flat.pdf

Ecological, evolutionary & environmental sciences study design

All studies must disclose on these points even when the disclosure is negative.

Study description	The data come from a 187 field trial of Genetically Modified, herbicide-tolerant (GMHT) crops in the UK (see Philosophical Transactions of the Royal Society (Issue 358, 2003). The trial was called the Farm Scale Evaluations of GMHT crops and made UK and worldwide headlines during the period 2000 - 2005. The FSEs is one of the largest agricultural experiments of management effects on biodiversity ever to be conducted. Each field was split in half (half-field design), with a conventional and GMHT variety of the same crop assigned to each half. 3 crops were tested (spring oilseed rape, beet and maize). The data analysed were year total data.
Research sample	The data analysed came from the pitfall sampling protocol for carabids, slug traps for molluscs, soil seedbank sampling for seed and the vegetation counts for standing plants. These protocols were part of sampling protocols for agricultural biodiversity used during the FSEs.
Sampling strategy	The protocols proved, in post-hoc power analyses of the power of the FSE experiment, to be sensitive protocols. Power analyses published in Clark et al. (2006). Proc. R. Soc. B 273, 237–243.
Data collection	The data were collected over a 3-agricultural year period from 2000 to 2005. Each field was followed for one agricultural year during this period, with approximately 1/3 of the 187 field sites being sampled at any one time. The large number of fields was split between research teams from three UK institutes (CEH, Rothamsted and SCRI). Please see Perry et al. (2003) Journal of Applied Ecology 40, 17–31. for a full description.
Timing and spatial scale	The data were year-total, national-scale data.
Data exclusions	No data were excluded.
Reproducibility	Reproducibility was analysed in the power analyses of the FSE, published as Clark et al. (2006). Proc. R. Soc. B 273, 237–243.
Randomization	Full randomization procedure for the trial were followed, with allocation of crops to field, and varieties to field halves following a completely (double blind) procedure as described in Perry et al. (2003) J. Appl. Ecol. 40, 17–31.
Blinding	All farmers involved in the study were initially unaware of which crop variety was allocated to each half of each field. All data were also anonymised to protect the farmers during this 'politically-charged' field experiment.

Did the study involve field work? ☒ Yes ☐ No

Field work, collection and transport

Field conditions	The field conditions were those of any large-scale agricultural field experiment. Field protocols had specific requirements for being conducted (temperature, rainfall, etc. see Philosophical Transactions of the Royal Society (Issue 358, 2003) for descriptions).
Location	The data were collected across the Great Britain national scale. A figure is provided in the Supplementary Materials.
Access & import/export	The field conditions were those of any large-scale agricultural field experiment. Field protocols had specific requirements for being conducted (temperature, rainfall, etc. see Philosophical Transactions of the Royal Society (Issue 358, 2003) for descriptions).
Disturbance	The field conditions were those of any large-scale agricultural field experiment. Field protocols had specific requirements for being conducted (temperature, rainfall, etc. see Philosophical Transactions of the Royal Society (Issue 358, 2003) for descriptions).

Reporting for specific materials, systems and methods

We require information from authors about some types of materials, experimental systems and methods used in many studies. Here, indicate whether each material, system or method listed is relevant to your study. If you are not sure if a list item applies to your research, read the appropriate section before selecting a response.

Materials & experimental systems

n/a	Involved in the study
☒	☐ Antibodies
☒	☐ Eukaryotic cell lines
☒	☐ Palaeontology and archaeology
☒	☐ Animals and other organisms
☒	☐ Human research participants
☒	☐ Clinical data
☒	☐ Dual use research of concern

Methods

n/a	Involved in the study
☒	☐ ChIP-seq
☒	☐ Flow cytometry
☒	☐ MRI-based neuroimaging
